# A case report of Chinese medicine combined with neoadjuvant chemotherapy in the treatment of human epidermal growth factor receptor 2 breast cancer

**DOI:** 10.1097/MD.0000000000043387

**Published:** 2025-08-15

**Authors:** Shiyuan Li, Shengliang Zhang

**Affiliations:** a Department of Gastrointestinal and Breast Surgery, Guizhou University of Traditional Chinese Medicine, Guiyang, Guizhou Province, China; b Department of Gastrointestinal and Breast Surgery, Guizhou University of Traditional Chinese Medicine, The First Affiliated Hospital of Guizhou University of Traditional Chinese Medicine of China, Guiyang, Guizhou Province, China.

**Keywords:** breast cancer, Chinese medicine, human epidermal growth factor receptor 2, neoadjuvant chemotherapy

## Abstract

**Rationale:**

According to Chinese medicine, surgical trauma and chemotherapy aggravate patients’ qi and blood deficiency and damage to the veins and channels. The combination of Chinese medicine and neoadjuvant chemotherapy (NACT) can not only improve the above symptoms, but also is expected to alleviate the adverse effects of breast cancer chemotherapy, such as nausea and vomiting, thereby improving patient compliance to achieve tumor reduction or even tumor-free, and providing a new diagnostic and therapeutic idea for the clinical treatment of breast cancer. This paper reported a case of human epidermal growth factor receptor 2 (HER-2) invasive breast cancer treated with traditional Chinese medicine combined with NACT. The lesion disappeared after 2 months of treatment and effectively reduced nausea and the symptoms of loss of appetite during NACT.

**Patient concerns:**

The patient was a female, 54-year-old, unintentionally found a double breast lump with tingling pain over 1 year. During this period, the patient did not pay attention to any treatment, the mass increased progressively, hard texture, unclear boundary. Denied family genetic history of breast cancer. The patient was diagnosed with invasive ductal carcinoma of both breasts (multiple metastases in right axillary and supraclavicular lymph nodes), T4N3M0 (tumour staging basis), stage IIC, HER-2 positive by breast needle biopsy. In the course of NACT, the patient complained of severe nausea and vomiting, which seriously affected the quality of life and chemotherapy confidence.

**Diagnoses:**

The patient was diagnosed with invasive ductal carcinoma of both breasts (multiple metastases in right axillary and supraclavicular lymph nodes), T4N3M0, stage IIC, HER-2 positive.

**Interventions:**

The patient was treated with NACT, and traditional Chinese medicine was taken orally.

**Outcomes:**

After 2 months of combination therapy, the breast mass shrank, the symptoms of nausea and vomiting were significantly relieved, and the appetite became better. The biopsy of the breast tissue cut at the completion of NACT showed no upper, lower, internal, outer, and basal lesions.

**Lessons:**

Chinese medicine internal effect is remarkable and safe, new adjuvant chemotherapy combined with Chinese medicine internal effectively improve nausea, vomiting symptoms, and effectively inhibit tumor hyperplasia, shrink tumor lesions, this case allows us consider whether in breast cancer new adjuvant chemotherapy combined Chinese medicine internal effect can be better, and provide new ideas for clinical treatment.

## 1. Introduction

Breast cancer is a phenomenon of uncontrolled proliferation of breast epithelial cells under the influence of multiple oncogenic factors, and neoadjuvant chemotherapy (NACT) has become an important tool in the comprehensive treatment of breast cancer. NACT is aimed at decreasing the tumor size and clinical stage, so as to improve the success rate of surgery and the survival rate of patients. However, the response to NACT varies among patients, which may be closely related to the molecular typing and immunohistochemical characteristics of breast cancer. Human epidermal growth factor receptor 2 (HER-2) type breast cancer accounts for about 20% to 25% of all breast cancers. This type of breast cancer is highly invasive and has a poor clinical prognosis. In this case, for the 1^st^ time, the combination of internal administration of traditional Chinese medicine (TCM) and double-targeted treatment of HER-2 type breast cancer achieved the effect of disappearance of the original tumor foci and no local or distant metastasis.

## 2. Case report

**Figure 1.**
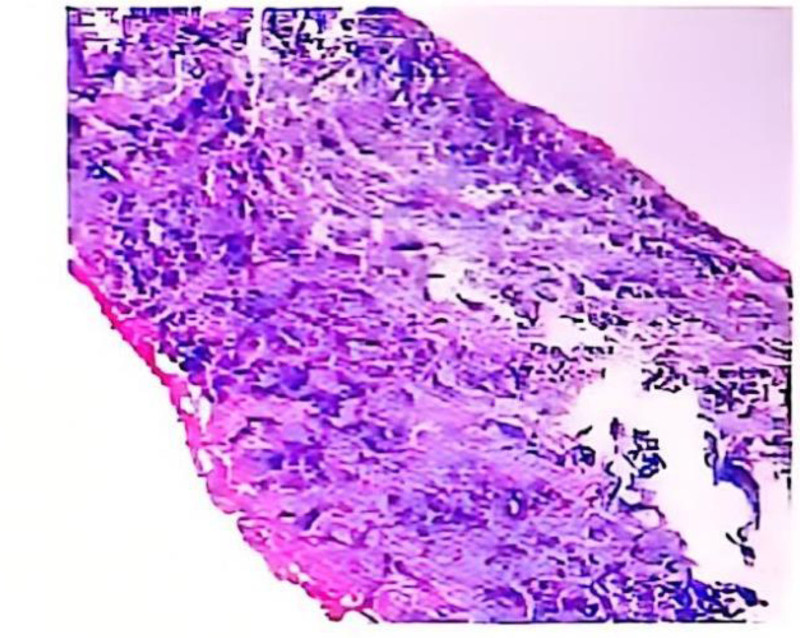
Malignant tumor of the left breast, combined with immunohistochemical findings supporting invasive ductal carcinoma of the breast (histological score 3 + 2 + 2 + 2 + 2 = 7, NGS classification: G2). NGS = next generation sequencing.

**Figure 2.**
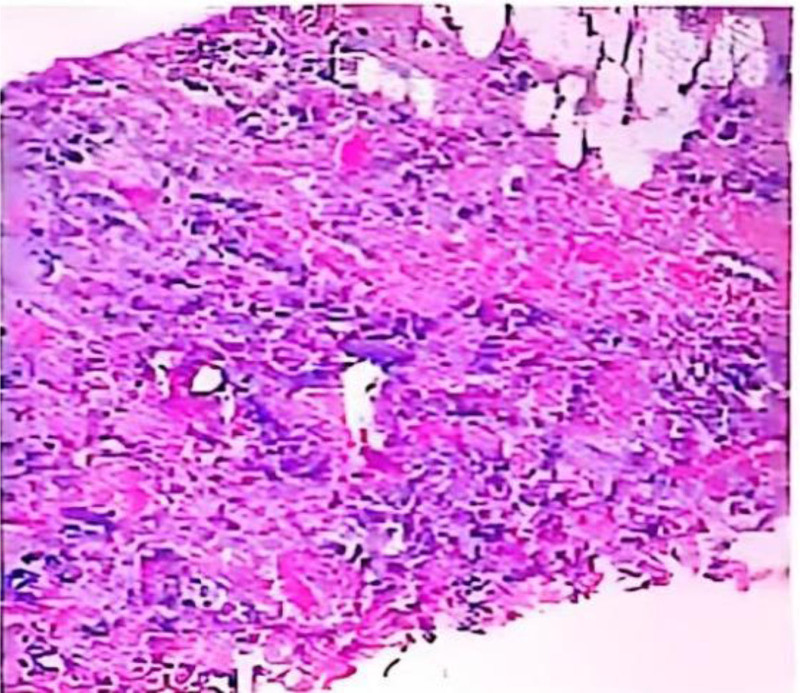
Malignant tumor of the right breast, combined with immunohistochemical results supporting invasive ductal carcinoma of the breast (histological score 3 + 2 + 2 + 2 + 2 = 7, NGS classification: G2). Immunohistochemical results showed: left tissue CK5/6(−), CK7(+), E-cadherin(+), ER(−), HER-2(+++), P63(−), Ri67(30%+), P120(+), PR(−); right tissue CK5/6(−),CK7(+), E-cadherin(+), ER(−), HER-2(+++), P63(−), Ri67(20%+), P120(+), P63(−), PR(−). ER = estrogen receptor, HER-2 = human epidermal growth factor receptor 2; NGS = next generation sequencing, PR = progesterone receptor.

The patient who was a female of 54-year-old unintentionally found a double breast lump with tingling pain for over 1 year. The left side was about 6 × 5 cm, the size of about 5 × 4 cm on the right side, hard texture, blurred boundary, occasional abdominal distension pain, chest tightness and discomfort, low mood, general appetite, thin white tongue coating, smooth pulse. The patient had a previous history of gastric ulcer, intermittent use of omeprazole enteric-coated tablets, a 2-year history of hepatitis B cirrhosis, and regular administration of entecavir. The breast ultrasound suggested large patchy hypoechoicity in the body layer of the right breast, Breast Imaging Reporting and Data System category 5, which was considered cancer, and the pathological results of the puncture were later suggested, malignant tumor of both breasts combined with the immunohistochemistry results and supported invasive ductal carcinoma of the breast, as shown in Figures 1 and 2, after completing relevant examinations, the diagnosis was clearly made as: invasive ductal carcinoma of both breasts (multiple metastases in right axillary and supraclavicular lymph nodes), T4N3M0, stage IIC, HER-2 positive, and it was proposed to carry out the neoadjuvant treatment of TCbHP (T stands for paclitaxel, Cb is for carboplatin, the H is for trastuzumab, and the P is for patulizumab). According to the patient’s height and body weight (50 kg, 156 cm, body surface area ≈ 1.47 m²), to balance efficacy and tolerability, the National Comprehensive Cancer Network guidelines recommend a starting dose of docetaxel at 75 mg/m², the specific regimen was as follows: (docetaxel: 110 mg + carboplatin: 595 mg) × 6 times + (trastuzumab 400 mg for the 1^st^ time, 300 mg for the 2^nd^, patuximab 840 mg for the 1^st^ time, 420 mg for the 2^nd^, for 1 year), 21 days and 1 cycle. The patient had fever, nausea, vomiting, bone marrow, suppression systemic itching, and erythema allergic reaction after the 1^st^ course of targeted therapy, which seriously affects the quality of life, combined with the National Comprehensive Cancer Network guideline suggests that the myelosuppression of carboplatin is more obvious, and if the patient develops an allergic reaction, the drug can be discontinued or replaced with a new treatment plan, so the removal of carboplatin adjusted the chemotherapy regimen for THP (T stands for docetaxel and HP is dual‐targeted therapy, i.e. trastuzumab and patuximab) (docetaxel: 110mg) × 5 times + (trastuzumab 400 mg for the 1^st^ time, 300 mg for the 2^nd^, patuximab 840 mg for the 1^st^ time, 420 mg for the 2^nd^, for 1 year). The patient’s symptoms include chest tightness and distension, heave a sigh, depression or irritability, insomnia and dreaminess, bitter taste in the mouth, low food intake, pain and abdominal distension and red tongue, thin yellow coating, and slippery pulse, who was given TCM according to the textbook of Chinese Medicine Surgery and Breast Cancer Treatment Guidelines 2023 (formula composition: 20 g of Gualou, 15 g of wine-washed Chaihu, 15 g of zhe beimu, 15 g of Xiangfu, 12 g of Yujin, 1 2g of Baishao, 10 g of Ruxiang, 10 g of Bamboo Ru, 10 g of Myrrh, and 5 g of Gancao). According to the meridian theory of TCM, the nipple and breast belong to the liver, gallbladder, and stomach meridians, nipple is the flush of the liver and kidney meridians, the breast is the place where yangming qi and blood converge, and the meridians and channels of the breast depend on the liver’s excretion and Yangming division. The occurrence of breast cancer is due to the injury of the liver and spleen, and the formation of phlegm and qi. When the liver is injured, the blood and qi stagnate in the breast; when the spleen is injured, the water and dampness are not transformed and become phlegm, and when the qi and phlegm are condensed, tuberculosis will gradually develop. The liver likes to be organized and is averse to depression. Liver qi is uncomfortable and stagnant in the chest and hypochondrium, and if it does not get through, it is painful, so it is seen as chest tightness, distension, and pain in the chest and hypochondrium, poor mood, and a liking for sighing. Gualou and Beimu dissolve phlegm to disperse knots, chaihu, xiangfu, and yujin detoxify the liver to relieve depression, Baishao softens the liver and relieves pain, ruxiang, bamboo ru, and myrrh move qi to smooth qi and arrest vomiting, and Gancao mediates all the herbs.

**Figure 3.**
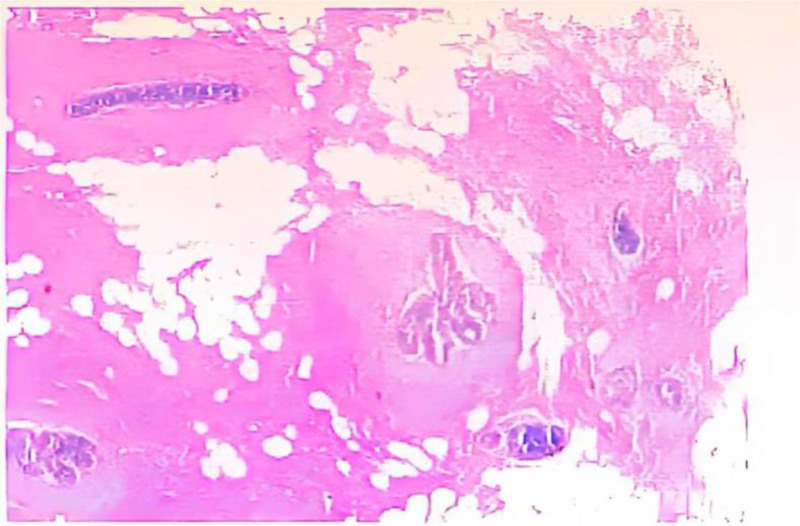
Left breast mass frozen pair and put away tissue sent for examination no ars tumefactive lesions in the tissue, MP grade after neoadjuvant chemotherapy for breast cancer: grade 5 (65). Tumorous lesions were seen in the upper, lower, inner, outer, and evaporative floor margins. Immunohistochemistry: PCK (+), CK5/6 (+), P63 (+). MP = Miller–Payne, PCK = pan‐cytokeratin.

**Figure 4.**
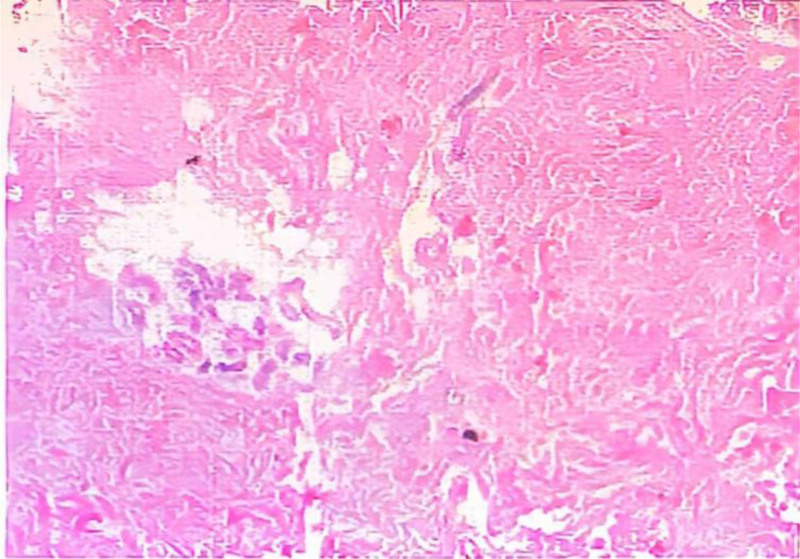
Tumourous lesions were seen by intraoperative surgery of the right breast mass frozen pair and frozen tissue sent for examination, MP grade after neoadjuvant chemotherapy for breast cancer: grade 5 (C5). No neoplastic lesions were seen in the superior, inferior, internal, external, or basal margins. MP = Miller–Payne, PCK = pan‐cytokeratin.

After 4 courses of chemotherapy and targeted therapy combined with TCM, the patient’s breast tumors had shrunk significantly, and the original right axillary and supraclavicular metastatic lymph nodes could not be touched. After completing 6 treatments, the patient underwent surgical resection and was sent for pathological examination, which showed the following: Frozen pair of the left breast mass and frozen tissue, no tumor lesion was seen in the tissue sent for examination, Miller–Payne classification after NACT for breast cancer: grade 5 (G5). No tumorigenic lesions were seen in the upper, lower, inner, outer, or basal margins. Immunohistochemistry: (section 15) pan‐cytokeratin (epithelial+). CK5/6 (myoepithelial+), P63 (myoepithelial+). Freeze-pairing of right breast mass and freeze-liquid tissues, no neoplastic lesions were seen in the sent tissues, Miller–Payne grading after NACT for breast cancer: grade 5 (G5). No tumorigenic lesions were seen in the upper and lower, inner, outer, and basal margins, as shown in Figures 3 and 4.

## 3. Discussion

Breast cancer has become the top malignant tumor affecting women’s health worldwide.^[1–3]^ The invasiveness and prognosis of breast cancer tumor cells are mostly related to the over expression of HER-2, and at present, it is recommended that patients with HER-2-type breast cancer be treated with long-term application of targeted drug therapy to achieve improvement of HER-2 metastatic breast cancer’s quality of life, and even have the effect of prolonging the survival expectation.^[4,5]^ Trastuzumab and patuzumab are a class of therapeutic drugs targeting HER-2, which mainly induce immune cells to exert toxic inactivation and inhibit cancer cell signaling through the tumor microenvironment, thus inhibiting the reproduction and metastasis of tumor cells, and the cancer cells ultimately undergo apoptosis as a result.^[6]^ When this patient was found to have multiple metastases in the right axilla and supraclavicular lymph nodes, the lymph nodes were palpable at that time with a size of 2 × 1 cm, hard texture and poor mobility. The original volume of the tumor was significantly reduced after 2 rounds of anti-HER-2-targeted therapy and chemotherapy combined with internal administration of TCM, and the nausea, vomiting, bone marrow suppression, and loss of appetite in the course of targeted therapy and chemotherapy were significantly reduced. After completing 6 treatments, because of the patient’s strong willingness for surgery, and considering that the volume of the tumor was significantly reduced after combined treatment with TCM, the patient was given the opportunity to have the primary fociresected, and the results of the resected foci sent for examination during the operation showed that there were no tumorous lesions in the breasts, and no metastasis of lymph nodes sent for examination in the breasts bilaterally; and the postoperative review of the chest computed tomography suggested that no abnormalities had been seen. The treatment plan of 1 patient with HER-2 breast cancer reported in this paper is the 1^st^ time to use the combination of: internal administration of TCM associated with HER-2 dual-targeted therapy and chemotherapy treatment, and also reflects the feasibility and safety of this plan. Early breast cancer is mainly manifested as asymptomatic lumps, which are described in ancient literature as small lumps and nodules. According to Chinese medicine, constitution represents the physiological and pathological characteristics of human beings as a whole, which not only determines the susceptibility and tendency of the disease, but also influences the regression and prognosis of the disease to a certain extent.^[7]^ The development of breast cancer is closely related to the liver and spleen, mainly manifested as liver depression and qi stagnation, and liver and spleen disorders. In addition, the occurrence of this disease is related to hormones, and it is found that people with qi depressive constitution with negative emotions for a long period of time are prone to endocrine imbalance and immunity decline, which promotes the development of tumors.^[8]^ Expert consensus suggests that the diagnosis and treatment of breast cancer mainly consists of 4 periods: perioperative period, perichemotherapy period, periradiotherapy period, and consolidation period,^[9]^ which are closely related to liver depression, yin deficiency and blood stasis; yin deficiency induces phlegm and stasis of blood, and turbid matter staying for a long period of time can be transformed into heat and toxicity, which leads to qi depression, phlegm condensation, and stasis of blood sticking to each other, so the treatment should be to support the positive qi, nourish yin and clear away the heat, dredge the liver to relieve depression and dissolve phlegm and remove stasis. The treatment is to support positive qi, nourish yin and clear heat, dredge the liver to remove depression, dissolve phlegm, and remove stasis. Chemotherapeutic drugs are hot and toxic in nature, easy to aggravate the loss of yin and fluid, qi and blood, qi and blood disharmony is not comfortable with the liver and spleen, the breast belongs to the liver, stomach meridian, qi is blocked, long-term stagnation of the breasts and thus the onset of the disease.

Modern research has found that the incidence rate, recurrence rate, and drug resistance rate of breast cancer in obese people are higher than that of normal weight patients, while the survival rate is the opposite.^[10]^ The TCM used in this paper is the plus–minus formula of Piper betel, in which the active ingredients of Piper betel (quercetin, kaempferol, and atractylenolide II) can be beneficial in preventing breast cancer via the nuclear factor E2-related factor/NAD(P)H:quinolone oxidoreductase pathway.^[11]^ Studies have shown that extracts of Phellodendron bidentata can effectively inhibit breast cancer cell viability and induce apoptosis.^[12]^ Chaihu mediated the inhibition of breast cancer tumor cell growth through the interleukin 12/toll-like receptor 4 signaling pathway.^[13]^ *Psidium guajava* was identified as a compound with anticancer ability, which can effectively reduce the chance of invasion and spread of tumor cells.^[14]^ Domestic studies on the use of Psoralea vulgaris Beisan are mostly in the postoperative period of breast cancer, and the main effects are to reduce postoperative lymph node edema, reduce the content of interleukin 8 and tumor necrosis factor alpha in the drainage fluid after breast cancer surgery, and regulate T lymphocytes.^[15]^ In addition, adverse chemotherapeutic reactions during chemotherapy should be included in the diagnosis and treatment plan during the treatment process, such as nausea and vomiting in the digestive system, drugs with the efficacy of harmonizing the stomach and stopping vomiting can be added as appropriate, such as Zhu Ru, and Xuan Fuhua. Thrombocytopenia and anemia can be appropriately added to nourish blood and activate blood, such as Chi Shao, Dang Gui, yolk of an egg. In the course of treatment, the patient complained of occasional nausea and vomiting, the degree of which was tolerable and did not affect the patient’s appetite and sleep. At the end of 6 cycles, the patient’s tumor lesions were significantly reduced compared with the previous ones and then underwent surgical resection, left breast mass frozen pair and put away tissue sent for examination no ars tumefactive lesions in the tissue, MP grade after neoadjuvant chemotherapy for breast cancer: grade 5 (65). Tumorous lesions were seen in the upper, lower, inner, outer, and evaporative floor margins. Immunohistochemistry: PCK (+), CK5/6 (+), P63 (+), and the postoperative pathology suggested that no tumor lesions were seen at the upper, lower, inner, outer, and basal edges of the bilateral breast subcutaneous lumps. The Chinese medicine effectively alleviated the adverse reactions of the digestive tract of the patient’s NACT and improved the patient’s compliance so as to complete 6 courses of NACT, but its specific pharmacological effects are still lack of specific clinical experiments to verify, and it may play a role in inhibiting cell proliferation, regulating apoptosis, and other aspects. It should be noted that this paper is a single case report, with a small and limited sample size, a lack of control, failure to further explore its mechanism of action, animal experiments, and multicenter randomized controlled trials to verify its effectiveness.

## 4. Conclusion

For patients with large primary tumors, strong willingness to preserve breasts, or patients with lymphatic metastasis, the combination of the herbal formula and NACT can reduce the size of the tumor, or even eliminate the tumor, and alleviate the adverse reactions of the chemotherapy process. The effect of the combination of Chinese herbal medicine and NACT can be used to reduce the size of the tumor, even eliminate the tumor, and alleviate the adverse reactions during chemotherapy.

Supplemental digital content is available for this article (https://links.lww.com/MD/P533).

## Acknowledgments

We thank patients for participating in this study.

## Author contributions

**Writing – review & editing:** Shiyuan Li, Shengliang Zhang.

## Supplementary Material


