# Supplementary figures and images for: A case report of Chinese medicine combined with neoadjuvant chemotherapy in the treatment of human epidermal growth factor receptor 2 breast cancer
Source: Medicine (Baltimore). 2025 Aug 15;104(33):e43387. doi: 10.1097/MD.0000000000043387 (PMC12366986; doi:10.1097/MD.0000000000043387)

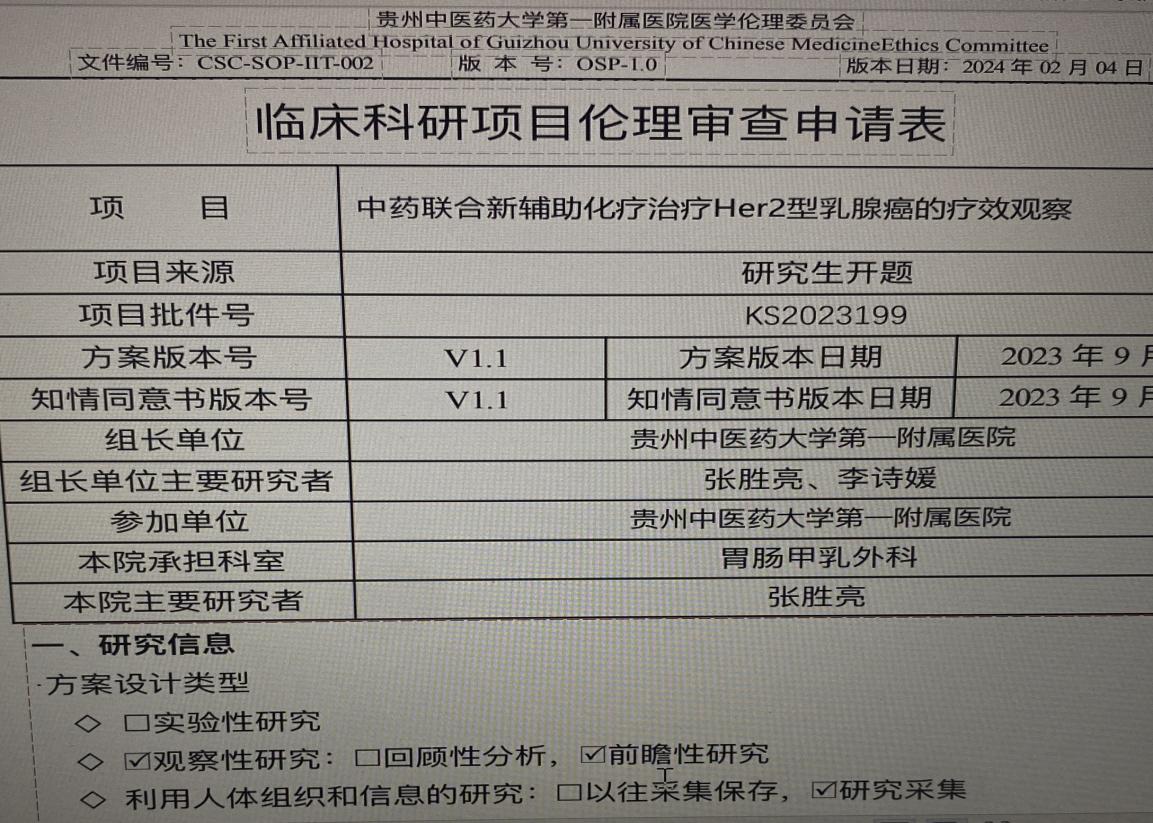

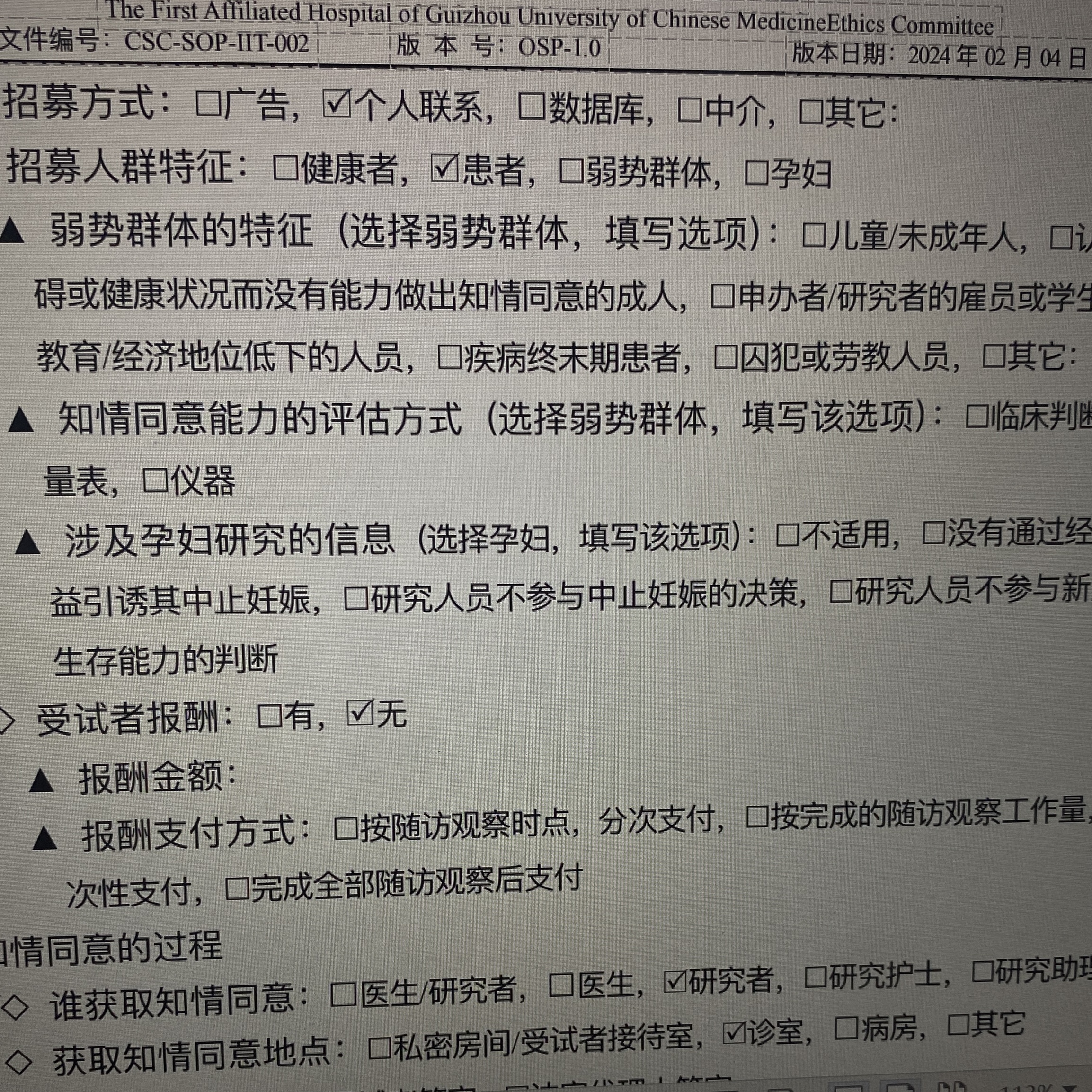

Supplement: Supplementary file 1 [file medi-104-e43387-s001.docx]
